# Suitability of Google Trends™ for Digital Surveillance During Ongoing COVID-19 Epidemic: A Case Study from India

**DOI:** 10.1017/dmp.2021.249

**Published:** 2021-08-03

**Authors:** Parmeshwar Satpathy, Sanjeev Kumar, Pankaj Prasad

**Affiliations:** 1 Department of Community Medicine, Veer Surendra Sai Institute of Medical Sciences and Research, Burla, Odisha, India; 2 Department of Community and Family Medicine, All India Institute of Medical Sciences (AIIMS), Bhopal, Madhya Pradesh, India

**Keywords:** disease surveillance, infodemiology, ICT in healthcare, pandemic, time lag correlation

## Abstract

**Objective:**

Digital surveillance has shown mixed results as a supplement to traditional surveillance. Google Trends™ (GT) (Google, Mountain View, CA, United States) has been used for digital surveillance of H1N1, Ebola and MERS. We used GT to correlate the information seeking on COVID-19 with number of tests and cases in India.

**Methods:**

Data was obtained on daily tests and cases from WHO, ECDC and covid19india.org. We used a comprehensive search strategy to retrieve GT data on COVID-19 related information-seeking behavior in India between January 1 and May 31, 2020 in the form of relative search volume (RSV). We also used time-lag correlation analysis to assess the temporal relationships between RSV and daily new COVID-19 cases and tests.

**Results:**

GT RSV showed high time-lag correlation with both daily reported tests and cases for the terms “COVID 19,” “COVID,” “social distancing,” “soap,” and “lockdown” at the national level. In 5 high-burden states, high correlation was observed for these 5 terms along with “Corona.” Peaks in RSV, both at the national level and in high-burden states corresponded with media coverage or government declarations on the ongoing pandemic.

**Conclusion:**

The correlation observed between GT data and COVID-19 tests/cases in India may be either due to media-coverage-induced curiosity, or health-seeking curiosity.

## Introduction

With more than 63000000 cases and more than 1400000 deaths reported worldwide by December 1 2020, the COVID-19 pandemic is the biggest human health threat since the deadly Influenza Pandemic of 1918.^1^ Countries across the globe have implemented various pharmacological and non-pharmacological control measures with mixed results. India responded with an initial travel restriction and thermal screening of inbound travelers in February 2020, which was followed by the largest population-level lockdown from 25 March, 2020 till 31 May, 2020.^2^

Surveillance of infectious diseases in India has been conducted through the Integrated Disease Surveillance Project (IDSP) network since 2004.^3^ Real-time surveillance was initiated in the form of Integrated Health Information Platform (IHIP) in 7 states in 2018.^4^ India’s health system consists of government-run health facilities and private health providers. Private health providers who cater to nearly 80% of ambulatory healthcare, do not contribute much to IDSP.^5,6^

People seek health information either from curiosity generated by the media, by virtue of their own, or a family member’s illness. Health information seeking, sometimes referred to as infodemiology has been suggested as another avenue of disease surveillance.^7^ Such ‘digital surveillance’ has been used during the previous H1N1, Ebola, Zika, and Chikungunya outbreaks.^8^ Researchers have analyzed the information- seeking behavior on Google™ Search Engine, Twitter™, and YouTube™ etc., to predict and explain infectious diseases outbreaks at the population level. Google Trends™ (Google, Mountain View, CA, United States) (GT) is an automated digital platform which provides data on Google search queries in form of Relative Search Volume (RSV).^9^ It has been used during epidemics of H1N1 influenza, Ebola Virus Disease, Dengue Fever, Zika, MERS, and Conjunctivitis etc., as well as for cancer screening, sinusitis, and scarlet fever, etcetera.^10–20^ With over 560000000 internet users in India, infodemiology can supplement IDSP.^21^ A study from India reported correlation between GT and IDSP reporting, in diseases such as Malaria, Dengue, Chikungunya and Enteric fever.^22^ Information- seeking on SARS-CoV-2 can similarly be used for digital surveillance and inform policy making for containment and mitigation. During the current COVID-19 pandemic, many studies have been published showing the usefulness of GT, including 2 from India.^23–34^ Most of these studies have not used comprehensive search strategy and used few COVID-19 related terms.^28^

We explored COVID-19 information-seeking-GT data from India to understand its utility in digital surveillance. We also used GT to correlate information-seeking on COVID-19 with the number of tests and cases reported both at national and state levels.

## Methods

### Data sources and search strategy

We obtained daily data on total cases and tests for India from the World Health Organization’s (WHO) COVID database, and covid19india.org website respectively. From the start of the pandemic till 31 May, 2020; 3737027 tests, 182143 cases, and 5164 deaths were reported in India.^35,36^ We obtained daily data on the total number of cases and tests for each of 5 high- and low- burden states from covid19india.org website on June 4, 2020. The highest number of cumulative cases were reported from the states/UTs of Maharashtra, Tamil Nadu, Delhi, Gujarat, and Rajasthan (67655, 22333, 19844, 16794, and 8831 respectively), while the lowest cumulative cases were reported by states/UTs of Sikkim, Mizoram, Daman and Diu, Arunachal Pradesh, and Meghalaya (1, 1, 2, 4, and 27 cases respectively). Cumulative tests for the 5 states/UTs with the highest caseloads were 463177, 491962, 212784, 211930, and 409777 respectively; while cumulative tests for the states/UTs with the lowest caseloads were 2985, 777, 11477, 8283, and 7781 respectively on May 31, 2020.

We explored Google Trends™ (Google, Mountain View, CA, United States) on June 3- 4, 2020. We included January 1, 2020 as the initial date, since the first report of the novel coronavirus pneumonia was reported from China on 31 December, 2019. We chose May 31, 2020 as the end-date to coincide with the notification of the end of lockdown in India. All data used in our study are open-source hence we did not seek explicit permission to utilize the data. We developed a list of 88 terms related to COVID-19, both in English and Hindi languages (Table 1) after discussion among the authors and other experts. We used both Roman and Devanagari scripts for the Hindi terms. We grouped these terms into 5 categories, and searched for these terms both individually and in groups to identify any trend during the study period. We could not find any trend for 21 and 49 terms respectively during the individual and group searches so we removed them and were left with 14 terms in English and 4 terms in Hindi for our final analysis (italicized in Table 1).

**Table 1.** Search strategy on google trends of covid-19 in India

Disease-related	Coronavirus, Corona, Covid 19, Covid, SARS-CoV-2, SARS Novel coronavirus, Novel corona, Virus, Infection, Disease
NPI and miscellaneous	Social distancing, Hand wash, Hand rub, Mask, Facemask, Sanitizer, Soap
Disease symptoms and treatment	Fever, Cough, Cold, Breathlessness, Fatigue, Rhinorrhoea, Nasal congestion, Sneeze, Myalgia, Sore throat, Diarrhoea, Anorexia, Chest pain, Headache, Nausea, Ageusia, Abdominal pain, Dizziness, Vomiting, Eye pain, Anosmia, Doctor, Nurse, Hospital, Clinic, Medicine, Check-up, OPD, Treatment, Testing
Government instructions & miscellaneous	Lockdown, Quarantine, Isolation, Bhilwada model, Curfew, Diya, Thali, Warrior, Shop, Market, Open, Ticket, Rail, Bus, Modi, PM cares, 20 lakh, Kerala, Mumbai
All searches for terms in Hindi script	*Khansi* (Cough), *Bukhar* (Fever), Dawa (Medicine), *Dawai* (Medicine), *Kharash* (Sore throat), *Sans* (Breathlessness), *Sardi* (Cold), *Jukam* (Cold), *Dama* (Asthma), coronavirus, Corona, Covid 19, Covid, Crona, Lockdown, Social distancing, *hath dhona* (Hand wash), *Mukhauta* (Mask), *Sabun* (Soap), *Deepak* (Lamp), *Thali* (Plate)

Abbreviations: NPI, Non-Pharmacological Interventions

We filtered the Relative search volume (RSV) data both at national and state levels. Search data from the Google Trend website were exported in.csv format and later converted into.xlsx format for analysis. The results were obtained in form of graphs and tables. RSV of ‘100’ suggested maximum search interest during the reference period while ‘0’ suggested no search interest for a particular term. *‘Peak’* generally referred to when RSV of 100 was observed for a particular term, while *‘spike*’ referred to any sharp rise followed by a fall.

### Correlation between GT data with daily tests and cases

We compared the GT data with daily data on COVID-19 tests and cases, both at national and the 10 selected state levels. We used Pearson’s correlation analysis to examine the correlations of RSV data of Google search terms with daily tests conducted, and daily new laboratory confirmed COVID-19 cases separately. We used the advanced data analysis tools available in Microsoft Excel 365 for this. We considered the correlation coefficient (r) of ≥ 0.5 as high and a *P* value of < 0.01 (Bonferroni’s procedure was used for correcting family wise error) as a statistically significant result. We used time lag correlation analysis to assess the temporal relationships for up to 14 days. The level of significance was set at 95%.

## Results

### Trend of disease-related terms (“coronavirus,” “corona,” “covid 19,” and “covid”)

The earliest search in India on Google for both “coronavirus” and “corona” was found on January 30, 2020. The first spike for these terms was visible on March 4, both at the national level and in high -burden states. It coincides with the first search for the terms “Covid 19” and “Covid.” Peak searches reflected as RSVs of 100 were observed for all 4 terms on March 23, and 28 at the national level, and March 28, and April 2 respectively for most of the states except for Tamil Nadu and Meghalaya. The maximum number of searches related to “Covid-19” was seen in the cities of Mira Bhayandar, Thane, Navi Mumbai, Mumbai, and Gurugram.

### Trends of non-pharmacological intervention (NPI) terms (“mask,” “sanitizer,” “social distancing,” “hand wash,” and “soap”)

**Figure 1.**
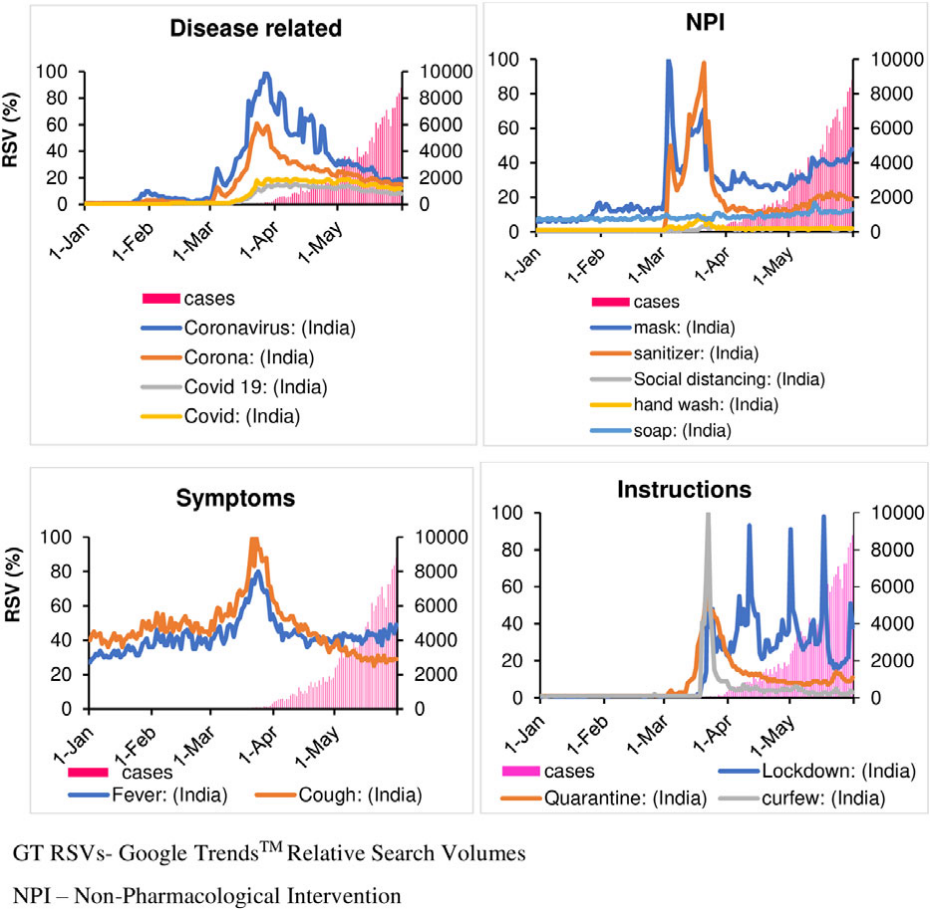
Time trend of COVID-19-related search terms in India with GT RSVs of the respective terms and daily covid-19 cases.

**Figure 2.**
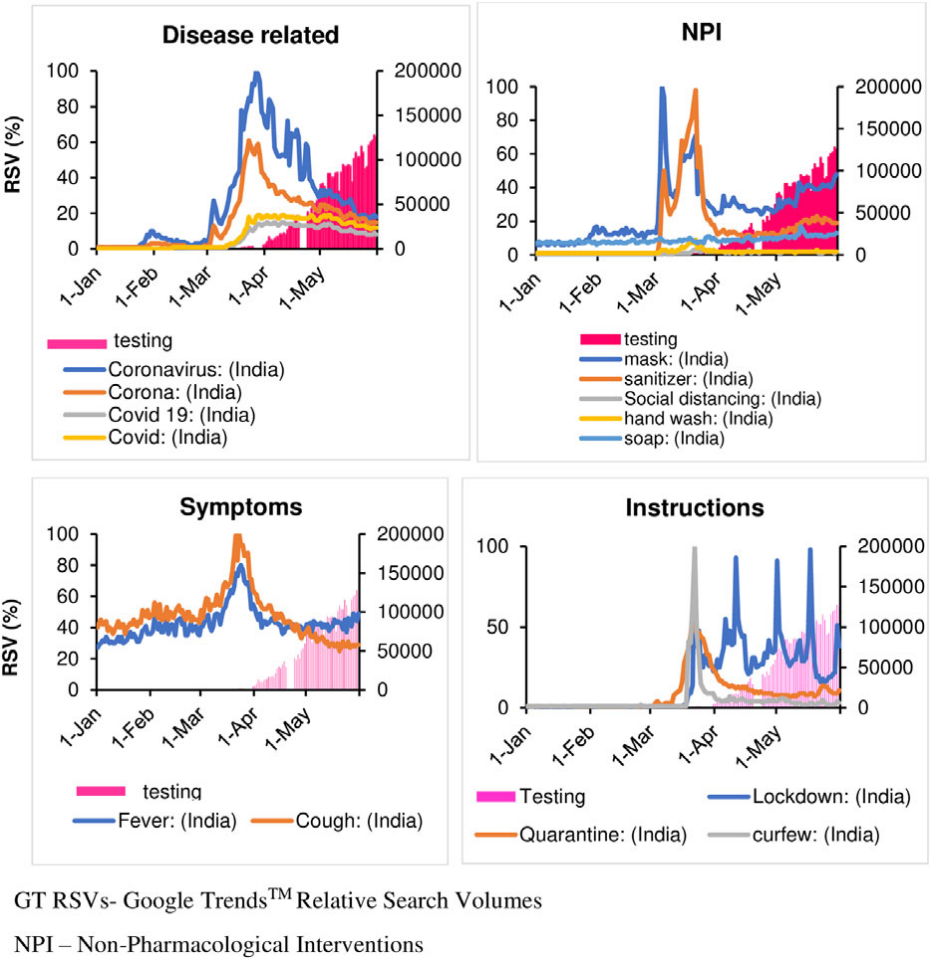
Time trend of COVID-19-related search terms in India with GT RSVs of the respective terms and daily COVID-19 testing.

Peak search for the term “Mask” happened on March 5, 2020 but the first spike was seen on 30 January 2020. The first spike for the term “sanitizer” was found on March 5. Afterwards, both the terms “mask” and “sanitizer” had mirrored trends with “mask” having a higher trend. Google searches for “social distancing,” “hand wash,” and “soap,” were very low throughout the observed period at both national and high-burden state levels (Figures 1 and 2). The states/UTs with the lowest caseloads did not have any trend for NPI terms.

### Trends for English terms for disease symptoms (“fever” and “cough”)

The terms “fever” and “cough” did not have distinct spikes before the peak. The peak search for “fever” happened on March 24, 2020 while that for “cough” occurred on March 23. Similar to Google Trends™ (Google, Mountain View, CA, United States) for disease related and NPI terms, “fever” and “cough” had similar results for states/UTs with high caseload. The states/UTs with the lowest caseloads had no clear trend, but had many crest-troughs with RSV often reaching 0.

### Trends for terms for government instructions (“lockdown,” “quarantine,” and “curfew”)

The first spike of search for “lockdown” happened on March 22, 2020 followed by 3 more spikes on April 11, May 1, and May 17, which coincides with announcement of lockdown by government of India. For “quarantine” and “curfew,” there were single peaks on March 22. Search trends for states/UTs with highest caseloads had similar trends at the national level. Among states/UTs with lowest caseloads, Meghalaya had a single peak for the term “curfew” on March 1 although the ‘junta curfew’ was declared on March 21. For Mizoram, 2 peaks were observed on March 1 and 5, 2020. For the term “quarantine,” Mizoram and Daman–and-Diu had their peaks on February 3 and 15, 2020 respectively. Sikkim had 4 peaks for the term “quarantine,” with the first being on February 9.

### Trends for Hindi terms for disease symptoms (“khansi,” “bukhar,” “sardi,” and “sans”)

The terms “khansi” and “bukhar” were also never at baseline before their peaks on March 21 and 22. Search for the term “sardi” started with a peak on January 1, 2020 and then tapered to remain between RSVs of 20 and 60 between February 1 and May 31, 2020. The term “sans” had RSV of 20 to 50 throughout the reference dates (Online supplementary file 3).

States/UTs with the highest caseloads had similar trends for the Hindi terms for disease symptoms, except Delhi which had multiple peaks and troughs without any trend. There was an insufficient number of searches for these terms in states/UTs with the lowest caseloads to show any trends.

### Correlation between GT data with daily tests and cases

High time-lag correlation was observed between both the daily number of tests as well as daily new laboratory-confirmed cases of COVID-19 with the Google search indices for the terms “COVID 19,” “COVID,” “social distancing,” “soap,” and “lockdown” at national level (Tables 2 and 3). The Pearson Correlation Coefficient was highest between “soap” and Covid-19 cases reported with a time lag of 14 days (r = 0.78, *P* < 0.00001). There were some differences in the trends of the results of the time lag correlation of NPI search terms and “lockdown” with daily new cases at national level. While “social distancing” and “soap” search terms showed high time-lag correlation with number of daily tests as well as cases for the entire time lag of 14 days, the other search terms such as “hand wash,” “mask,” and “lockdown,” showed comparatively low time-lag correlation during the period (Tables 2 and 3).

**Table 2.** Time-lag correlations between google search terms and daily new laboratory-confirmed covid-19 cases for India

Lag (days)	Coronavirus	Corona	Covid 19	Covid	Mask	Sanitizer	Social distancing	Hand wash	Soap	Lockdown	Quarantine	Bukhar	Sans
0	0.02	0.14	0.45	0.49	0.35	0.09	0.46	0.03	**0.78**	0.43	0.05	−0.04	0.30
1	0.03	0.15	0.46	**0.50**	0.34	0.09	0.47	0.04	**0.77**	0.43	0.05	−0.01	0.32
2	0.04	0.17	0.47	**0.51**	0.33	0.09	**0.50**	0.04	**0.77**	0.43	0.05	−0.01	0.36
3	0.05	0.18	0.49	**0.53**	0.32	0.09	**0.50**	0.04	**0.78**	0.44	0.05	0.02	0.38
4	0.07	0.19	**0.50**	**0.54**	0.32	0.09	**0.50**	0.04	**0.78**	0.45	0.06	0.04	0.42
5	0.09	0.21	**0.52**	**0.56**	0.31	0.08	**0.50**	0.04	**0.77**	0.48	0.07	0.03	0.42
6	0.10	0.22	**0.54**	**0.57**	0.31	0.08	**0.51**	0.04	**0.79**	**0.50**	0.07	0.05	0.43
7	0.12	0.23	**0.55**	**0.59**	0.30	0.07	0.50	0.05	**0.79**	**0.52**	0.07	0.07	0.42
8	0.14	0.25	**0.56**	**0.60**	0.29	0.08	**0.53**	0.05	**0.81**	**0.53**	0.07	0.09	0.46
9	0.15	0.26	**0.58**	**0.61**	0.29	0.07	**0.56**	0.05	**0.80**	**0.55**	0.06	0.10	0.48
10	0.17	0.28	**0.60**	**0.63**	0.28	0.07	**0.59**	0.06	**0.81**	**0.58**	0.06	0.13	0.48
11	0.19	0.29	**0.61**	**0.65**	0.28	0.07	**0.59**	0.05	**0.80**	**0.60**	0.07	0.12	0.49
12	0.21	0.31	**0.63**	**0.66**	0.27	0.07	**0.59**	0.05	**0.77**	**0.63**	0.08	0.13	0.45
13	0.23	0.32	**0.65**	**0.67**	0.27	0.07	**0.60**	0.06	**0.78**	**0.64**	0.09	0.14	0.47
14	0.25	0.33	**0.66**	**0.68**	0.26	0.06	**0.60**	0.07	**0.78**	**0.63**	0.09	0.14	0.45

Values in bold text shows high correlation with *r* ≥ 0.5 at a statistical significance *P*-value of 0.01

**Table 3.** Time-lag correlations between google search terms and daily new covid-19 tests in India

Lag (days)	Coronavirus	Corona	Covid 19	Covid	Mask	Sanitizer	Social distancing	Hand wash	Soap	Lockdown	Quarantine	Bukhar	Sans
0	0.04	0.17	**0.50**	**0.54**	0.34	0.08	**0.50**	0.03	**0.81**	0.49	0.04	0.00	0.34
1	0.05	0.18	**0.52**	**0.56**	0.33	0.08	**0.52**	0.04	**0.82**	0.49	0.04	0.03	0.36
2	0.06	0.19	**0.53**	**0.57**	0.32	0.08	**0.54**	0.04	**0.80**	0.49	0.04	0.01	0.39
3	0.07	0.20	**0.55**	**0.59**	0.31	0.07	**0.53**	0.04	**0.79**	**0.51**	0.04	0.04	0.40
4	0.09	0.22	**0.57**	**0.61**	0.31	0.07	**0.53**	0.03	**0.77**	**0.54**	0.05	0.07	0.40
5	0.11	0.24	**0.58**	**0.62**	0.30	0.06	**0.53**	0.03	**0.76**	**0.55**	0.05	0.08	0.42
6	0.13	0.25	**0.60**	**0.63**	0.29	0.06	**0.53**	0.04	**0.78**	**0.55**	0.06	0.08	0.41
7	0.15	0.27	**0.61**	**0.65**	0.28	0.05	**0.54**	0.04	**0.79**	**0.55**	0.06	0.10	0.41
8	0.17	0.28	**0.63**	**0.66**	0.27	0.05	**0.57**	0.05	**0.79**	**0.55**	0.06	0.10	0.45
9	0.19	0.30	**0.64**	**0.68**	0.26	0.05	**0.59**	0.05	**0.79**	**0.56**	0.06	0.10	0.47
10	0.22	0.31	**0.66**	**0.69**	0.26	0.05	**0.61**	0.06	**0.78**	**0.59**	0.07	0.13	0.48
11	0.25	0.33	**0.68**	**0.71**	0.25	0.04	**0.62**	0.05	**0.75**	**0.62**	0.08	0.15	0.47
12	0.28	0.35	0.70	0.72	0.25	0.04	0.63	0.05	0.73	0.65	0.09	0.18	0.47
13	0.30	0.37	0.71	0.73	0.24	0.04	0.64	0.06	0.74	0.67	0.10	0.19	0.48
14	0.32	0.38	0.72	0.74	0.24	0.05	0.64	0.07	0.75	0.65	0.12	0.19	0.46

Values in bold text show high correlation with *r* ≥ 0.5 at a statistical significance *P*-value of 0.01

**Figure 3.**
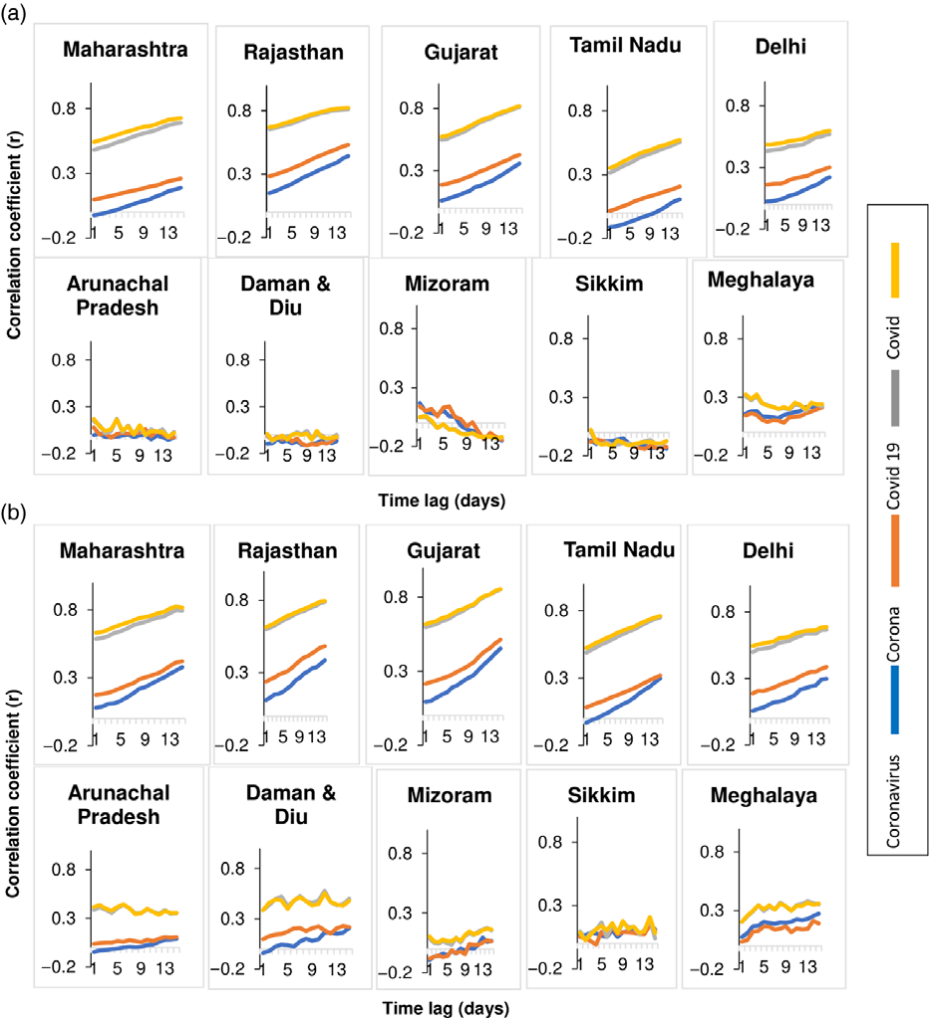
Lag correlations between google search terms related to disease with (a) daily new laboratory-confirmed covid-19 cases, and (b) daily new tests in various states of India.

**Figure 4.**
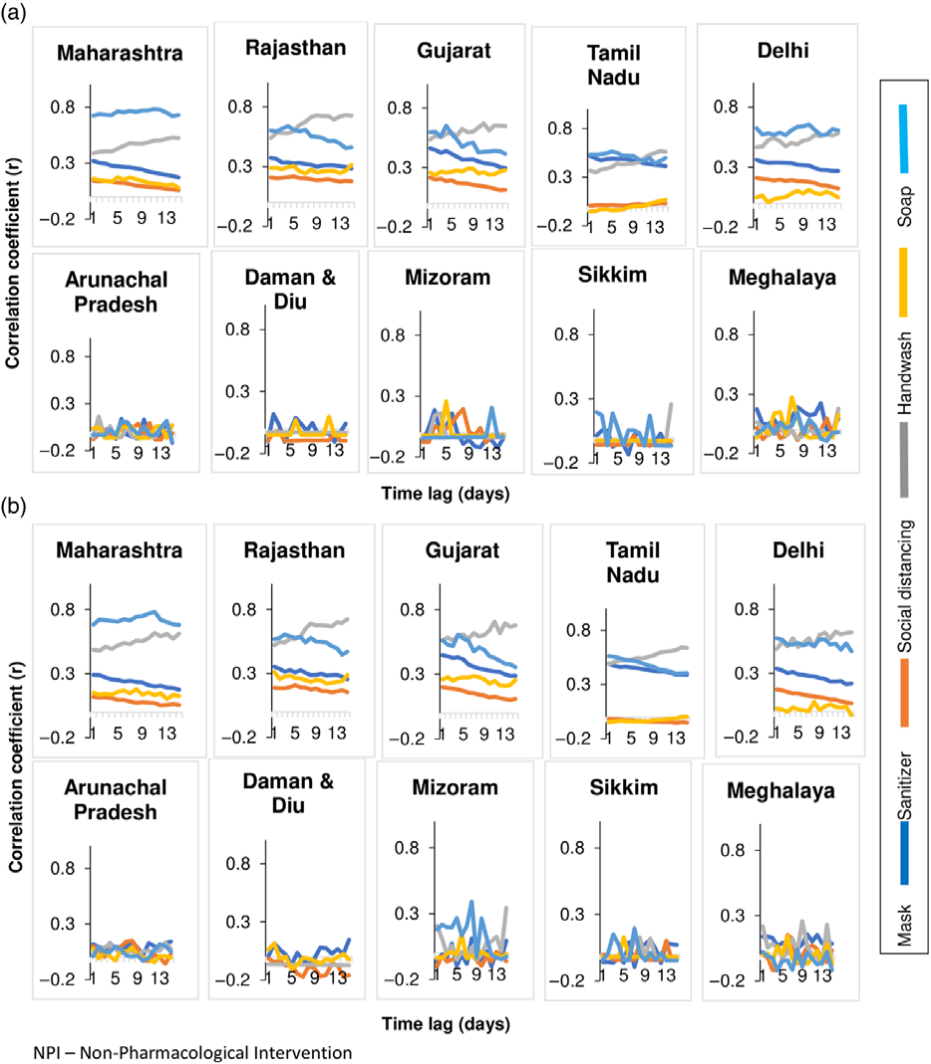
Lag correlations between Google search terms related to NPI with (a) daily new laboratory-confirmed covid-19 cases, and (b) daily new tests in various states of India.

High time-lag correlation was observed between both the daily number of tests as well as daily new laboratory-confirmed cases of COVID-19 with the Google search indices for the terms “COVID 19,” “COVID,” “Corona,” “social distancing,” “soap,” and “lockdown” for the 5 high-burden states (Figures 3 and 4). In contrast, for the low burden states, there was no time-lag correlation (Figures 3 and 4). Highest correlation of the term “soap” was seen with tests and cases in states/UTs of Maharashtra, Rajasthan, Gujarat, Tamil Nadu, and Delhi. The time-lag correlation between the term “soap” and the number of daily tests was 0 days for both Tamil Nadu and Delhi. Rajasthan and Gujarat states had high time-lag correlations respectively with daily new laboratory-confirmed cases at 14 days (r = 0.53, *P* < 0.00001 and r = 0.51, *P* < 0.00001). Maharasthra and Delhi had similar high time-lag correlation with cases and the term “Fever” at 2 days (r = 0.518, *P* < 0.00001), and 0 days (r = 0.56, *P* < 0.00001) respectively.

## Discussion

We analyzed search behavior on the Google search engine for Coronavirus related information with the Google Trends (Google, Mountain View, CA, United States) tool for India, as well as 10 states with highest and lowest caseloads. However, we could not identify any published studies using similar comprehensive search strategy or including GT data from states/UTs of India.^33,34^ For other countries, researchers have used only a few terms for Google Trend analysis of Covid-19 online search behaviour.^24–27,29–32^ We found 2 studies which reported the use of various methods to arrive at a detailed search strategy, but they have not reported the details.^28,42^ Categorization of the search terms is expected to answer 2 assumptions. First, the search behavior may simply reflect information-seeking out of curiosity, and apprehension among citizens generated by pandemic coverage by the media. Our search categories of disease terms, NPI terms, and government instruction terms is expected to reflect curiosity-induced search behavior. A study conducted to evaluate reliability of Google Trends™ (Google, Mountain View, CA, United States) for digital surveillance reports the influence of media coverage.^43^ COVID-19 infodemiology studies have also reported similar GT data correlation of COVID-19 disease related terms with media coverage.^44^ A stark difference is seen in the United States of America (USA), where peak interest was reported on March 9 and 12, coinciding with the issuance of interim guidelines and the declaration of Novel Coronavirus pandemic by World Health Organization (WHO).^23^ In India, search trends did not peak on these 2 dates however, as Indian mainstream mass media did not report these developments. Secondly, people seek health information to guide themselves following actual illness episodes in self or family members. The second assumption in search behavior would augment disease surveillance activities by providing a prediction of outbreaks earlier than traditional methods. Google Trends (Google, Mountain View, CA, United States) had been argued to have earlier predictive utility for H1N1 epidemic, dengue fever, Chikungunya, and Malaria, etc.^12,22,45–47^ Moreover, our study included GT data for a longer duration, unlike most of the other studies reporting Google Trend analysis for COVID-19 infodemiology.

The earliest search for the terms “coronavirus” and “corona” in India likely reflects media reports of the first confirmed case. The earliest spikes for disease-related terms likely coincides with media reports of the first cluster of cases reported among Italian tourists from Rajasthan.^48^ The largest peak in the search for disease-related terms coincides with the declaration of ‘junta (citizen) curfew’ and lockdown by the Indian Prime Minister in a televised national address which was widely reported by various sections of the media.^49,50^ The same analogy should apply individually to the 10 states except Meghalaya. Cities with maximum searches related to “Covid-19” have the highest population densities in India and the highest caseloads in the initial phase of the pandemic. This may indicate true health-seeking behavior. COVID-19-related searches in India slowly began to decline after the last week of March, 2020 most likely due to flooding of information from various print, electronic and social media leading to information fatigue and disinterest.

NPI terms that we have used in our study have been used by few other studies as well for GT analysis.^26,30,32,41^ The first search spike for these terms is likely related to the first case reported from Kerala, India. Searches for the term “Mask” in India reached 2 peaks much earlier than that for disease related search terms. Like disease related terms, the first peak coincides with the report of the first cluster of COVID-19 cases among Italian tourists. The second peak coincides with observation of citizen curfew likely reflecting curiosity. We argue that such health-information seeking may not correlate with healthcare seeking in India. This is in contrast with similar searches in internet-savvy countries during influenza epidemics.^51^ Our assumption is buttressed by the finding of poor trends for search behavior pertaining to 3 other NPI terms: “social distancing,” “hand wash,” and “soap.” Previous studies have shown that people in India are mostly aware of the effectiveness of handwashing with soap as healthy behavior, while the use of masks and social distancing are considered to be useful during epidemics like H1N1.^52,53^ While handwashing has been shown to be consistently increasing, “Masks” and “sanitizers” have been infrequently used community-level preventive measures in Indian health system till the current COVID-19 pandemic. The increased search interest for “Mask” and “sanitizer” is also linked to the listing of these items under the essential commodity list by the Indian government.^54^ In states with low caseloads, users were more interested in searching about sanitizers than masks.

Finally, the lesser interest in search for the symptoms (specifically “fever” and “cough”) both in English and Hindi languages strongly supports our argument that the search behavior in India was mostly a result of curiosity rather than health-seeking. Only a few similar studies from India have reported the use of these search terms in their analysis.^33^ Globally, we also identified only few published studies which used “fever” to analyze Google Trends™ results during the ongoing COVID-19 pandemic.^28^ Google Trends™ analysis using the term “fever” has previously been reported for other diseases such as dengue fever and Influenza.^12,55,56^ Coexistence of Influenza and COVID-19, or Dengue fever and COVID-19 cannot be ruled out only by search behavior using this term during winter and rainy seasons respectively. For the term “cough,” we also could not identify any published study on Covid-19 from India using Google Trends™ analysis. Few other studies have used “cough” for Google Trends™ analysis in diseases like Sinusitis and COVID-19.^19,28,44^ The peak trends for “fever” and “cough” in India coincided with the declaration of the lockdown and with peak search trends for coronavirus-disease related terms and NPI terms. Similar peak trends in high-burden states reflect the curiosity following the lockdown declaration.

Regarding trends for terms related to government instructions (“lockdown,” “quarantine,” and “curfew”), peaks for “lockdown” both at the national level and for the high-burden states coincides with each of the 5 phases of the lockdown enforced by the Indian government. We argue that the only peak for “quarantine” and “curfew” is due to the curiosity generated through the observation of “junta (citizen) curfew.” The highest trend for the term “curfew” seen in Meghalaya and Mizoram states were due to local non-health issues.

We could not find any study from either India or other countries using Hindi terms related to COVID-19 for Google Trends™ analysis. Nearly 43.6% of Indian population uses Hindi as their first language.^57^ The term “sardi” which is the Hindi translation for both “common cold,” and “winter” had its peak on January 1 which coincides more aptly with the cold conditions of winter in India. This trend reflects media coverage for the extremely cold conditions in many parts of the country. The peaks for the terms “khansi” and bukhar” coincided with the Junta curfew, hence reflecting curiosity again. Using these terms may not help in the prediction of an outbreak through info-veillance.

This study showed high time-lag correlations by 10-14 days between the search behavior for Coronavirus related keywords using Google Trends™ and the number of new daily confirmed COVID-19 cases as well as tests. Shin *et al.,* in their study on Middle East Respiratory Syndrome (MERS) using Google and Twitter keywords have showed similar results with daily cases and quarantined cases.^13^ Various other studies have reported high time-lag correlation between disease related search terms and the increase in cases during an outbreak.^12,13,24,25,27,28,32,33^ The correlation was observed mainly for the terms “COVID 19,” “COVID,” “social distancing,” “soap,” and “lockdown” at the national level. Based on the available data, we are not sure whether these correlations are really due to information-seeking for healthcare or simply reflect curiosity. We have just explained that most of the peaks in search behavior for the 18 terms used for Google Trends™ analysis can be explained by curiosity emanating from media coverage of events like the declaration of lockdown, clustering of cases, etc. Community-based studies are required to ascertain the exact cause of health-information seeking.

This shows increased interest among the general population not only towards COVID disease related search terms such as “COVID 19” and “COVID” but also towards their own safety as observed by the high time-lag correlation throughout the period of 14 days for the terms “social distancing” and “soap.”

A high positive correlation between the number of infected cases and the Google trend values for the term “COVID 19” has been reported for India as part of the analysis for 8 major countries.^33^ Similarly, high positive time-lag correlation between the number of infected cases and the Google trend values was seen for the term “COVID” in USA.^42^ While the term “Coronavirus” showed high time-lag correlation in several studies with cases, our study did not have such correlation. This might be due to the fact that these studies were done between January and March, 2020 when the term “COVID 19” was not used widely.^24,27,32^ Increased apprehension of the population on whether the “lockdown” will end or whether it will be extended can be observed by the high time-lag correlation observed with daily cases at 14 days and with the daily tests at 13 days at the national level in India.

“Soap” was the term with the highest Pearson correlation coefficient at a time-lag of 10 days when analyzed against the actual daily COVID-19 cases and a time-lag of 1 day when analyzed against the daily COVID-19 tests at the national level. This might be due to either majority of population being well informed about the need for, and method of handwashing, or not giving due importance to handwashing over the use of other NPIs such as “sanitizer.” We hypothesize that media coverage of stock out of sanitizer might have prompted people to give more importance to sanitizer as compared to soap as a preventive method in COVID-19 epidemic. There were government instructions on including sanitizers in the list of essential commodities, which might further have deflected interest from soap to sanitizer. Therefore, we propose that “sanitizer” may be a more appropriate term for digital surveillance of COVID-19 outbreaks in India as compared to “soap” or “handwashing.” The high time-lag correlation observed in the high-burden states for these terms likely contributes to the observation at national level. These findings should form part of digital surveillance at state levels. It would be interesting to see whether such trends are seen in hitherto low-burden states which may develop outbreaks in future. The high correlation with the term “Fever” seen in the states of Maharashtra and Delhi is most likely predictive for an outbreak during the ongoing “COVID 19” epidemic in these states unlike the national level. This is in contrast to the results obtained by Higgins *et al.,* who reported positive correlation between Google search for symptoms of COVID-19 and the COVID-19 cases at level of countries.^28^

These findings reflect increase in internet search activities almost 10 to 14 days before the increase in daily COVID-19 cases and deaths. These findings indicate that Google Trends™ can serve 2 purposes. First, it can be used as a tool to monitor public restlessness toward COVID-19 infections in India to decide the timing and location for risk communication by governments. Such timely and focused risk communication can help avoid “infodemics” of panicky health-seeking. Second, it should be further explored whether prediction of emergence and propagation of COVID-19 outbreaks by 2 weeks by GT can supplement the traditional field-based surveillance mechanism. Previous epidemics have supported the use of internet searches for digital outbreak surveillance, pointing towards the fact that the digital surveillance deserves greater investment by the public health agencies.^14,58^ The advantages of using a tool such as Google Trends™ (Google, Mountain View, CA, United States) is that data can be obtained earlier, more easily, and at a much lower cost than routine surveillance techniques adopted by the governments. A study has compared the surveillance platform of India known as Integrated Disease Surveillance Project (IDSP) and reported strong correlation with a time-lag of 2 to 3 weeks for Chikungunya, Dengue Fever, Malaria and Enteric Fever.^22^ Similar studies should be attempted for COVID-19 data from IDSP and Google Trend™. Longitudinal studies need to be conducted among internet users to identify and follow-up their online search behavior related to COVID-19 epidemic to verify the reason(s) for such searches. Such studies can provide a correction factor which can be used while using GT for complementing the traditional surveillance system. We hypothesize that the lack of such confirmatory studies led to demolishing of Google Flu search network.^59^

Our study does have certain limitations. The selection of the keywords as well as the associated spelling might affect the overall results of the study. There is no globally accepted guideline for Google Trends™ (Google, Mountain View, CA, United States) analysis, though a procedure has been recommended by some authors.^7^ A guideline is required from Google™ in this regard due to their custody of the search data, but none- sharing of the algorithm for search.^9^ We did not have access to media coverage in India during this period to explore the correlation between COVID-19 related media coverage and GT data.

## Conclusion

This study reveals the advantages of info-veillance and infodemiology using Google Trends™ (Google, Mountain View, CA, United States) to monitor an emerging infectious disease like COVID-19 in India. The correlation observed between Google Trends^TM^ (Google, Mountain View, CA, United States) data with COVID-19 tests and cases in India may be due to search behavior induced either by media-coverage of the pandemic or health-seeking for COVID-19 illness. We argue that GT can supplement the traditional surveillance system, more easily and at a lower cost.

## Data Availability

All relevant data are within the paper and its Supporting Information files.

## Supplementary material

For supplementary material accompanying this paper visit http://dx.doi.org/10.1017/dmp.2021.249. click here to view supplementary material
