# Supplementary figures and images for: Suitability of Google Trends™ for Digital Surveillance During Ongoing COVID-19 Epidemic: A Case Study from India
Source: Disaster Med Public Health Prep. 2021 Aug 3:1–10. doi: 10.1017/dmp.2021.249 (PMC8460424; doi:10.1017/dmp.2021.249)

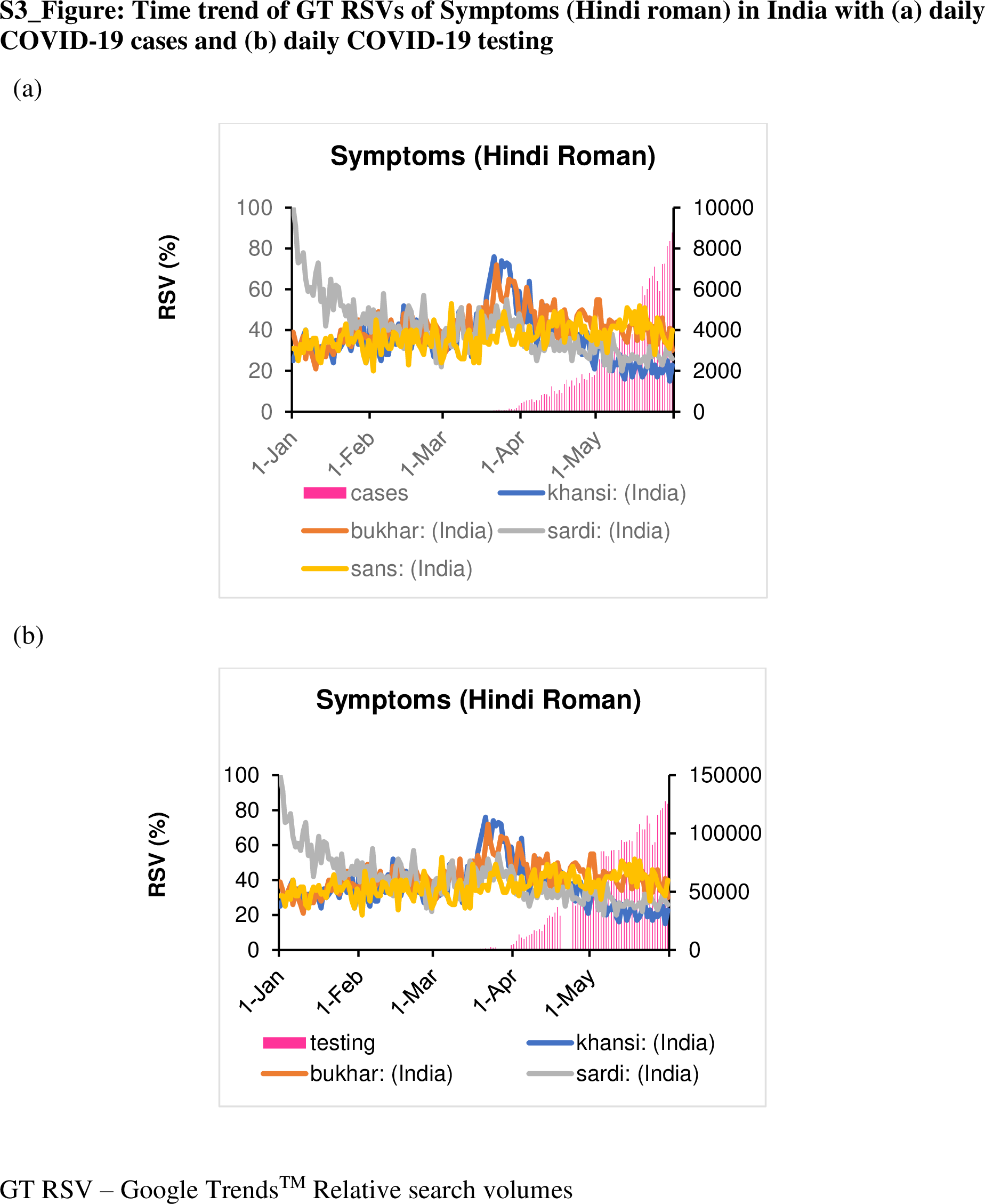

Supplement: Supplementary file 1 [file dmpsup.zip › S1935789321002494sup002.tif]
